# Complete mitochondrial genome of golden conure (*Guaruba guarouba*)

**DOI:** 10.1080/23802359.2016.1247670

**Published:** 2017-01-17

**Authors:** Adam Dawid Urantówka, Tomasz Strzała, Paweł Mackiewicz

**Affiliations:** a Department of Genetics, Wroclaw University of Environmental and Life Sciences, Wrocław, Poland;; b Department of Genomics, Faculty of Biotechnology, Wrocław University, Wrocław, Poland

**Keywords:** Psittaciformes, *Arini*, mitogenome, *Guaruba guarouba*, golden conure

## Abstract

*Arini* tribe with 19 genera is the most diversified tribe of neotropical parrots. Six of them are classified as macaws and nine as conures. The presence of bare facial area distinguishes macaws from conures and other members of this tribe. However, such morphological division seems to be disputable as the smallest macaw (monotypic *Diopsittaca* genus) turned out to be more closely related to three monotypic conures genera (*Guaruba*, *Leptosittaca*, *Thectocercus*) than to other macaws. We sequenced the complete mitochondrial genome of *Guaruba guarouba* to enrich the resource of molecular markers for examination of phylogenetic relationships between macaws and conures.

Subfamily *Arinae* (the New World parrots) is the most species-rich group within the order *Psittaciformes* (Schweizer et al. 2014). It is divided into four tribes (Schodde et al. 2013) from which the *Arini* tribe is the most taxon-rich. The majority of nineteen extant genera recognized within this tribe are divided into two morphologically diverse groups. Six of them (*Anodorhynchus, Ara, Cyanopsitta, Diopsittaca, Orthopsittaca* and *Primolius*) are classified as macaws (Forshaw 2010) based on the presence of bare facial area, which distinguishes them from other members of the tribe. Another nine genera (*Aratinga, Enicognathus, Eupsittula, Guaruba, Leptosittaca, Ognorhynchus, Psittacara, Pyrrhura* and *Thectocercus*) belong to conures (Remsen et al. 2016).

Recent molecular studies showed that a conure *Aratinga acuticaudata* should be shifted to a new genus *Thectocercus acuticaudatus* because it is more closely related to three monotypic genera (*Diopsittaca, Guaruba* and *Leptosittaca*) than to any member of the previously broadly defined genus *Aratinga* (Remsen et al. 2013; Urantowka et al. 2013). The correctness of macaws morphological classification was further undermined by the significant separation of the smallest macaw (*Diopsittaca nobilis*) from other members of this groups and its close relationship to conures – *Guaruba*, *Leptosittaca* and *Thectocercus*.

Many phylogenetic relationships within conures and macaws are still unsolved. Therefore, more molecular data are required to reconstruct precise their phylogenies. It was shown that complete mitochondrial genomes can provide useful information for evolutionary studies of many taxa (Nabholz et al. 2013). So far, complete mitogenomes of only three representative macaws (*Ara*, *Orthopsittaca* and *Primolius*) and four conures (*Eupsittula*, *Psittacara*, *Pyrrhura* and *Thectocercus*) are available (Pacheco et al. 2011; Urantowka et al. 2013; Urantowka et al. 2014; Urantowka 2016a,b,c; Urantowka et al. 2016). Therefore, we sequenced *Guaruba guarouba* mitogenome with the length of 17,008 bp (GeneBank accession number JQ782217) to gain appropriate molecular data for future examination of evolutionary diversification of macaws and conures.

Although morphology of the analyzed specimen (Polish captive bird with CITES document no. 1281/2009 issued on 15.07.2009 in Hannover) was absolutely typical for *Guaruba guarouba* individuals, we aligned its control region sequence with all available other such sequences of the species as well as selected macaws and conures, to prove its species belonging. The obtained tree (Figure 1) revealed that the analyzed *G. guarouba* individual grouped significantly with two other representatives of this species. The clade *Guaruba* was sister to the group of *Thectocercus* and *Diopsittaca*. Three macaws (*Ara*, *Orthopsittaca* and *Primolius*) formed a monophyletic group but were clearly separated from the smallest macaw (*Diopsittaca nobilis*).

**Figure 1.**
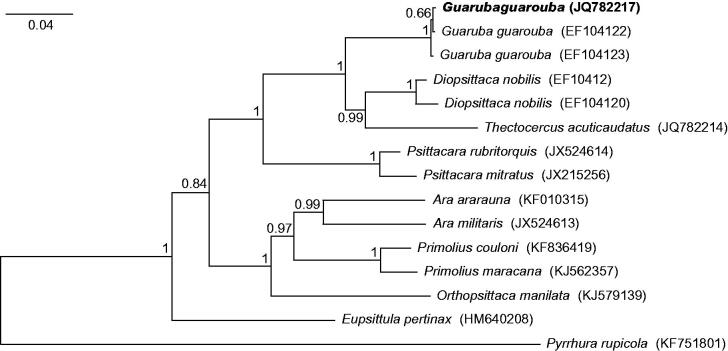
The phylogenetic tree obtained in MrBayes for control region sequences indicating that the studied individual (bolded) belongs to *Guaruba guarouba* species. The tree was generated with Bayesian method in MrBayes 3.2.5 (Ronquist et al. 2012) using the model GTR + I + G as suggested by jModelTest 2.1.7 (Guindon & Gascuel 2003; Darriba et al. 2012). 10,000,000 MCMC repetitions with burn-in of 25% was assumed. Tree was rooted with *Pyrrhura rupicola* sequence. Genbank accession numbers are shown in parenthesis. Bayesian posterior probabilities are shown at nodes.

Gene order found in *G. guarouba* mitogenome was the same as in *Thectocercus acuticaudatus* mitogenome, so far, the only fully sequenced member of the clade *Guaruba/Leptosittaca/Thectocercus/Diopsittaca*. The sequence identity of both genomes was 93.9% and base composition of their H-strand was nearly the same. The start and stop codons usage was also consistent for both species with the exception to the *atp6* gene. In *Guaruba guarouba,* this gene had a truncated (TA_) stop codon, but in *Thectocercus* it ended with TAA codon.

## References

[CIT0001] Darriba D, Taboada GL, Doallo R, Posada D. 2012 jModelTest 2: more models, new heuristics and parallel computing. Nat Methods. 9:772. 10.1038/nmeth.2109 PMC4594756 22847109

[CIT0002] Forshaw JM. 2010 Parrots of the World. London: A & C Black Publishers Ltd; p. 328.

[CIT0003] Guindon S, Gascuel O. 2003 A simple, fast, and accurate algorithm to estimate large phylogenies by maximum likelihood. Syst Biol. 52:696–704. 14530136 10.1080/10635150390235520

[CIT0004] Nabholz B, Uwimana N, Lartillot N. 2013 Reconstructing the phylogenetic history of long-term effective population size and life-history traits using patterns of amino acid replacement in mitochondrial genomes of mammals and birds. Genome Biol Evol. 5:1273–1290. 23711670 10.1093/gbe/evt083 PMC3730341

[CIT0005] Pacheco MA, Battistuzzi FU, Lentino M, Aguilar RF, Kumar S, Escalante AA. 2011 Evolution of modern birds revealed by mitogenomics: timing the radiation and origin of major orders. Mol Biol Evol. 28:1927–1942. 21242529 10.1093/molbev/msr014 PMC3144022

[CIT0006] Remsen JV Jr, Areta JI, Cadena CD, Jaramillo A, Nores M, Pacheco JF, Pérez-Emán J, Robbins MB, Stiles FG, Stotz DF, Zimmer KJ. 2016 A classification of the bird species of South America. American Ornithologists' Union; [version 2016 Sept 20]. Available from: http://www.museum.lsu.edu/∼Remsen/SACCBaseline.html

[CIT0007] Remsen JV, Schirtzinger EE, Ferraroni A, Silveira LF, Wright TF. 2013 DNA-sequence data require revision of the parrot genus Aratinga (Aves: Psittacidae). Zootaxa. 3641:296–300. 26287088 10.11646/zootaxa.3641.3.9

[CIT0008] Ronquist F, Teslenko M, van der Mark P, Ayres DL, Darling A, Höhna S, Larget B, Liu L, Suchard MA, Huelsenbeck JP. 2012 MrBayes 3.2: efficient Bayesian phylogenetic inference and model choice across a large model space. Syst Biol. 61:539–542. 22357727 10.1093/sysbio/sys029 PMC3329765

[CIT0009] Schodde R, Remsen JV Jr, Schirtzinger EE, Joseph L, Wright TF. 2013 Higher classification of new world parrots (Psittaciformes; Arinae), with diagnoses of tribes. Zootaxa. 3691:591–596. 26167605 10.11646/zootaxa.3691.5.5

[CIT0010] Schweizer M, Hertwig ST, Seehausen O. 2014 Diversity versus disparity and the role of ecological opportunity in a continental bird radiation. J Biogeogr. 41:1301–1312.

[CIT0011] Urantowka AD. 2016a Complete mitochondrial genome of critically endangered blue-throated macaw (*Ara glaucogularis*): its comparison with partial mitogenome of Scarlet macaw (*Ara macao*). Mitochondrial DNA A DNA Mapp Seq Anal. 27:422–424. 24621219 10.3109/19401736.2014.898287

[CIT0012] Urantowka AD. 2016b Complete mitochondrial genome of Blue-headed Macaw (*Primolius couloni*): its comparison with mitogenome of Blue-throated Macaw (*Ara glaucogularis*). Mitochondrial DNA A DNA Mapp Seq Anal. 27:2106–2107. 25391030 10.3109/19401736.2014.982578

[CIT0013] Urantowka AD. 2016c Complete mitochondrial genome of Red-bellied Macaw (*Orthopsittaca manilata*): its comparison with mitogenome of Blue-throated macaw (*Ara glaucogularis*). Mitochondrial DNA A DNA Mapp Seq Anal. 27:2110–2111. 25391028 10.3109/19401736.2014.982580

[CIT0014] Urantowka AD, Grabowski KA, Strzała T. 2013 Complete mitochondrial genome of Blue-crowned Parakeet (*Aratinga acuticaudata)-phylogenetic position of the species among parrots group called Conures*. Mitochondrial DNA. 24:336–338. 23351080 10.3109/19401736.2012.760080

[CIT0015] Urantowka AD, Kroczak AM, Strzała T. 2014 Complete mitochondrial genome of endangered Socorro conure (*Aratinga brevipes*) - taxonomic position of the species and its relationship with green conure. Mitochondrial DNA. 25:365–367. 23815322 10.3109/19401736.2013.803095

[CIT0016] Urantowka AD, Strzała T, Grabowski KA. 2016 The first complete mitochondrial genome of *Pyrrhura* sp.-question about conspecificity in the light of hybridization between Pyrrhura molinae and Pyrrhura rupicola species. Mitochondrial DNA A DNA Mapp Seq Anal. 27:471–473. 24660930 10.3109/19401736.2014.900672

